# A new genus and a new species in the subfamily Polyzosteriinae (Blattodea, Blattidae) from China

**DOI:** 10.3897/zookeys.852.33325

**Published:** 2019-06-05

**Authors:** Shuran Liao, Zongqing Wang, Yanli Che

**Affiliations:** 1 Institute of Entomology, College of Plant Protection, Southwest University, Beibei, Chongqing 400715, China

**Keywords:** Blattaria, cockroaches, *
Laevifacies
*, *
Melanozosteria
*, molecular identification, morphology

## Abstract

*Laevifaciesquadrialata***gen. et sp. nov.** is described from Hainan Province, China based on morphological data. COI data (DNA barcodes) is utilized to confirm the sexual dimorphism occurring in *Laevifaciesquadrialata***gen. et sp. nov.***Melanozosterianitida* Brunner von Wattenwyl, 1865, is reported from Guangxi Province, China. A key to the Chinese Polyzosteriinae is provided.

## Introduction

Polyzosteriinae is a relatively species-abundant subfamily in the Blattidae. The subfamily is flightless (except Methanini), having lobiform vestigial tegmina or being totally apterous. Some species have pits or tubercles scattered on the pronotum (Rentz 2014), short tarsi and large pulvilli and arolia (Mackerras 1968b). Members of Polyzosteriinae were firstly mentioned by Burmeister (1838), with the establishment of genus *Polyzosteria* Burmeister, 1838. Tepper (1893) erected the subfamily Polyzosteriinae with two genera, *Polyzosteria* and *Platyzosteria* Brunner von Wattenwyl, 1865. The revisionary works of Mackerras (1965a, 1965b, 1965c, 1966a, 1966b, 1967a, 1967b, 1968a, 1968b) included 16 genera, e.g. *Polyzosteria* Burmeister, 1838; *Platyzosteria* Brunner von Wattenwyl, 1865; *Cosmozosteria, Melanozosteria, Zonioploca* Stål, 1874; *Methana* Stål, 1877; *Anamesia, Drymaplaneta, Leptozosteria, Pseudolampra* and *Temnelytra* Tepper, 1893; *Desmozosteria, Euzosteria* and *Scabina* Shelford, 1909; *Eppertia* Shaw, 1925 and *Megazosteria* Mackerras, 1966a. In the catalogue, Princis (1966) recorded *Eurycotis* Stål, 1874. McKittrick (1964) provided a detailed description of the genitalia and proventriculus of the Polyzosteriinae female and male for the first time. Mackerras (1965a) divided the Australian members into two tribes: Polyzosteriini and Methanini, compiling the most complete account of Polyzosteriinae to date. Grandcolas (1997) described 5 genera from New Caledonia. Up to now, Polyzosteriinae contains 22 genera and 305 species (Beccaloni 2014), most of which are distributed in Australia, Southeast Asia, America, and the Pacific Islands.

The genus *Melanozosteria* was established with *Polyzosterianitida* Brunner von Wattenwyl, 1865 as type species (Stål 1874). After that, Mackerras (1967b) did not agree with *Melanozosteria* as a synonym of *Platyzosteria* owing to the misidentification of one species of *Platyzosteria* by Shelford (1909), and placed *Melanozosteria* and *Leptozosteria* in *Platyzosteria* as subgenera. Roth (2003) re-established the taxonomic status of *Melanozosteria* as a genus. Currently 44 species are known of *Melanozosteria*, which are mainly distributed in Australia (Beccaloni 2014). Two *Melanozosteria* species are currently recorded in China (*Melanozosterianitida* Brunner von Wattenwyl, 1865 and *Melanozosteriasoror* Brunner von Wattenwyl, 1865). *Melanozosterianitida* from Taiwan was originally determined as *Periplanetapolita* Walker, 1868. Then Shelford (1909) proposed that *Periplanetapolita* is a synonym of *Cutilianitida* Brunner von Wattenwyl, 1865. Until now, they have both been considered synonyms of *Melanozosterianitida*. In the catalogue, Princis (1966) recorded *Melanozosterianitida* from Taiwan, China, but he questioned its distribution on Mainland China. The other species, *Melanozosteriasoror*, is mainly distributed in Australia and the Pacific Islands. Walker (1868) firstly recorded this species from Taiwan, China (it was originally described as *Periplanetaphilpotti*, but later synonymized under *Melanozosteriasoror* in Princis (1957)). Then Shiraki (1931) recorded this species from Hainan, but no further information was provided.

DNA barcodes have been proven to be a helpful method to identify species and to successfully match male and female. Barcoding has been applied to resolve the problems of sexual dimorphism and even to identify nymphs in cockroaches (Evangelista et al. 2013; Qiu et al. 2017; Che et al. 2017; Wang et al. 2018). To date, members of the Polyzosteriinae have been identified primarily on the basis of morphological characters (Mackerras 1965a, 1965b, 1965c, 1966a, 1966b, 1967a, 1967b, 1968a, 1968b; Rentz 2014) and DNA Barcoding has not been employed to investigate the diversity of Polyzosteriinae. In this paper, *Laevifaciesquadrialata* gen. et sp. nov. is described from China and the sexual dimorphism is revealed via DNA barcoding. We also record a specimen from Guangxi, thus proving that *Melanozosterianitida* is also distributed in Mainland China. A key to the known Polyzosteriinae species from China is provided.

## Materials and methods

### Morphological study

Morphological terminology used in this paper mainly follows McKittrick (1964), Mackerras (1965a) and Roth (2003). Measurements are based on specimens examined. Genital segments of the examined specimens were macerated in 10% NaOH for 20 minutes and rinsed with distilled water, observed in glycerin jelly using a MOTIC K400 stereomicroscope. Photographs of the specimens were taken using a Canon® 50D plus a Canon® EF 100mm f/2.8L IS USM Macro lens combined with Helicon Focus® software. Photos of other characters were taken using a Leica® M205A stereomicroscope. All photographs mentioned above were modified in Adobe Photoshop® CS6. The type materials are deposited in the Institute of Entomology, College of Plant Protection, Southwest University, Chongqing, China.

### DNA extraction, PCR, and sequencing

We used two cockroach specimens for COI sequencing in this study in order to resolve the sexual dimorphism. Both sequences are deposited in GenBank with the accession numbers: MK798103, MK798104 (Table 1). The extraction procedure was according to the Hipure Tissue DNA Mini Kit (Magen Biotech, Guangzhou). Fragments of COI were amplified using PCR. Primers for the amplifications are LCO1490 (5’-GGTCAACAAATCATAAGATATTGG-3’) and HCO2198 (5’-TAAACTTCAGGGTGACCAAAAAATCA-3’) (Folmer 1994). The amplification conditions were: initial denaturation at 94 °C for 3 min, followed by 35 cycles for 30 s at 94 °C, 30 s at 49 °C, and 1 min at 72 °C, with a final extension of 10 min at 72 °C.

**Table 1.** Species used in this study

**Family**		**Species**	**Accession number**	**Reference**
Outgroups	Mantidae	* Mantis religiosa *	KM529415	Hebert et al. 2015 (Unpublished)
		* Mantis religiosa *	KR148854	Hebert et al. 2016
Ingroups	Blattidae	*Laevifaciesquadrialata* sp. nov.	MK798103	
		*Laevifaciesquadrialata* sp. nov.	MK798104	
		* Periplaneta australiasiae *	KX640825	Ma et al. 2017
		* Shelfordella lateralis *	KU684413	Cheng et al. 2016
		* Neostylopyga rhombifolia *	KP986425	Legendre et al. 2015
		* Hebardina concinna *	KF640073	Yue et al. 2014
		* Methana parva *	KP986422	Legendre et al. 2015
		* Angustonicus lifou *	KP986393	Legendre et al. 2015

### Sequence processing and phylogenetic analyses

A total of ten COI sequences were analyzed (two sequences of *Laevifacies* species from our study, six sequences of Blattidae, and two sequences of a mantid outgroup downloaded from GenBank) (Table 1). All COI sequences were aligned using MEGA 7.0 and adjusted visually after translation into amino acid sequences. Finally, for the phylogenetic analysis we acquired COI sequences whose lengths were 658 bp, except for *Angustonicuslifou* whose sequence was only 650 bp. The genetic divergence value was quantified based on the Kimura 2-parameter (K2P) distance model (Kimura 1980), using MEGA 7 (Kumar et al. 2016) with 1000 bootstrap replicates. Maximum Likelihood (ML) analysis was implemented in RAxML 7.3.0 (Stamatakis et al. 2008) using GTRGAMMA model with 1000 bootstrap replicates.

## Results

### Phylogenetic analysis based on COI

In this study, we acquired two COI sequences, whose length, excluding primers, was 658 bp each. The genetic divergence value between male and female of *Laevifaciesquadrialata* sp. nov. is 0.9%; however, the interspecific K2P genetic divergence among *Laevifaciesquadrialata* sp. nov. and other species ranged from 10.4 to 13.1%.

**Figure 1.**
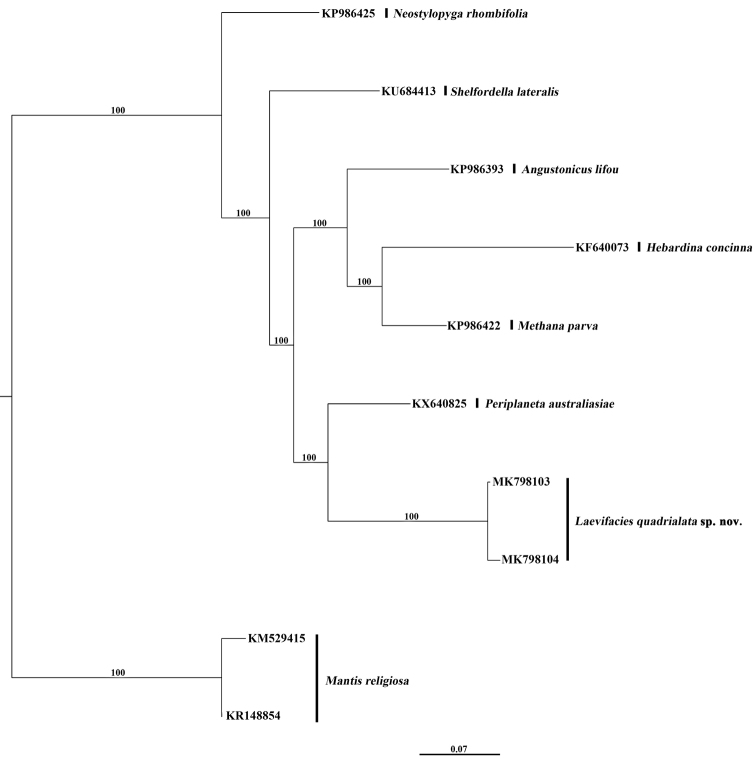
Maximum likelihood (ML) tree derived from COI gene analysis with 1000 bootstrap replicates. Number above branch indicates MLB.

The ML phylogenetic tree (Figure 1) revealed that male and female of *Laevifaciesquadrialata* sp. nov. grouped together with a high support value (MLB = 100).

## Taxonomy

### Subfamily Polyzosteriinae Tepper, 1893

Polyzosteriinae Tepper, 1893: 32; Princis 1950: 170; Princis 1960: 447; Princis 1966: 561; McKittrick 1964: 66; Mackerras 1965a: 841; Rentz 2014: 121.

### Key to Species of Polyzosteriinae in China

**Table:** 

1	Sexual dimorphism present. Body small; tegmina and hind wings vestigial in male; tegmina vestigial and hind wings absent in female	***Laevifaciesquadrialata* sp. nov.**
–	Sexes similar. Body large; tegmina vestigial and hind wings absent	**2**
2	Terga and abdomen uniformly dark reddish brown to black	*** Melanozosteria nitida ***
–	Margin of terga with continuous and broad yellow stripes, the middle black; sometimes abdomen with continuous or discontinuous yellow stripes	*** Melanozosteria soror ***

#### Laevifacies gen. nov.

Taxon classificationAnimaliaBlattodeaBlattidae

http://zoobank.org/EC93B8A9-1413-4EB1-B139-63AF641FD6E3

##### Type species.

*Laevifaciesquadrialata* sp. nov. here designated.

##### Generic diagnosis.

Body small to medium, thinner in male, thorax slightly broader than abdomen. Surface smooth and shining. Pronotum slightly semicircular, vertex barely exposed. Male with vestigial tegmina and hind wings on mesonotum and metanotum respectively, both nearly triangular; female only with vestigial tegmina, its shape similar to that of male, without hind wings. Legs strong but short, coxae with punctation, front femora Type A_2_. Mid and hind metatarsus with strong spines, claws symmetrical. Cerci strong, short and symmetrical. Styli long and symmetrical. Supra-anal plate in male short, triangular; subgenital plate broad and short, slightly quadrilateral and symmetrical. L1 divided into two parts, L3 bifurcated, one branch short, the other one long, R1 nearly claw-like and R2 large, hooked.

##### Etymology.

The name *Laevifacies* is derived from two Latin words *laevis* and *facies*, referring to the smooth and shining surface of terga. The gender of *Laevifacies* is feminine.

##### Remarks.

**Figure 2.**
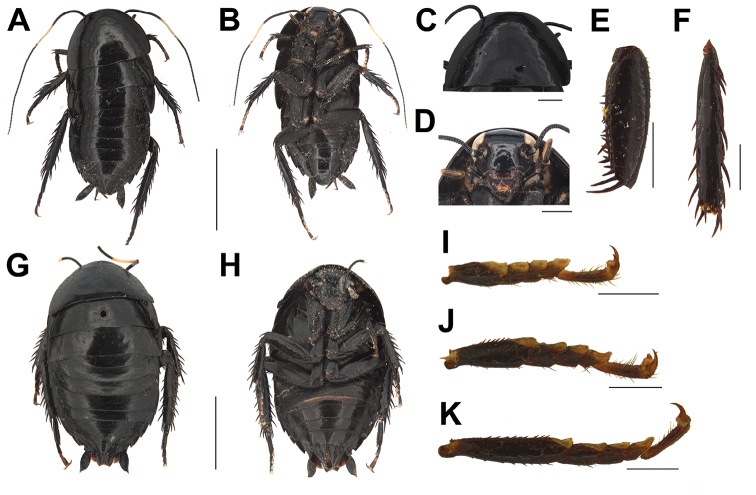
**A–K***Laevifaciesquadrialata* sp. nov. **A–F, I–K** male holotype **A** in dorsal view **B** in ventral view **C** pronotum, in dorsal view **D** head, in ventral view **E** femur, in ventral view **F** tibia, in ventral view **I** fore tarsus, in ventral view **J** mid tarsus, in ventral view **K** hind tarsus, in ventral view. **G–H** female paratype **G** in dorsal view **H** in ventral view. Scale bars: 5 mm (**A–B, G–H**); 1 mm (**C–F, I–K**).

**Figure 3.**
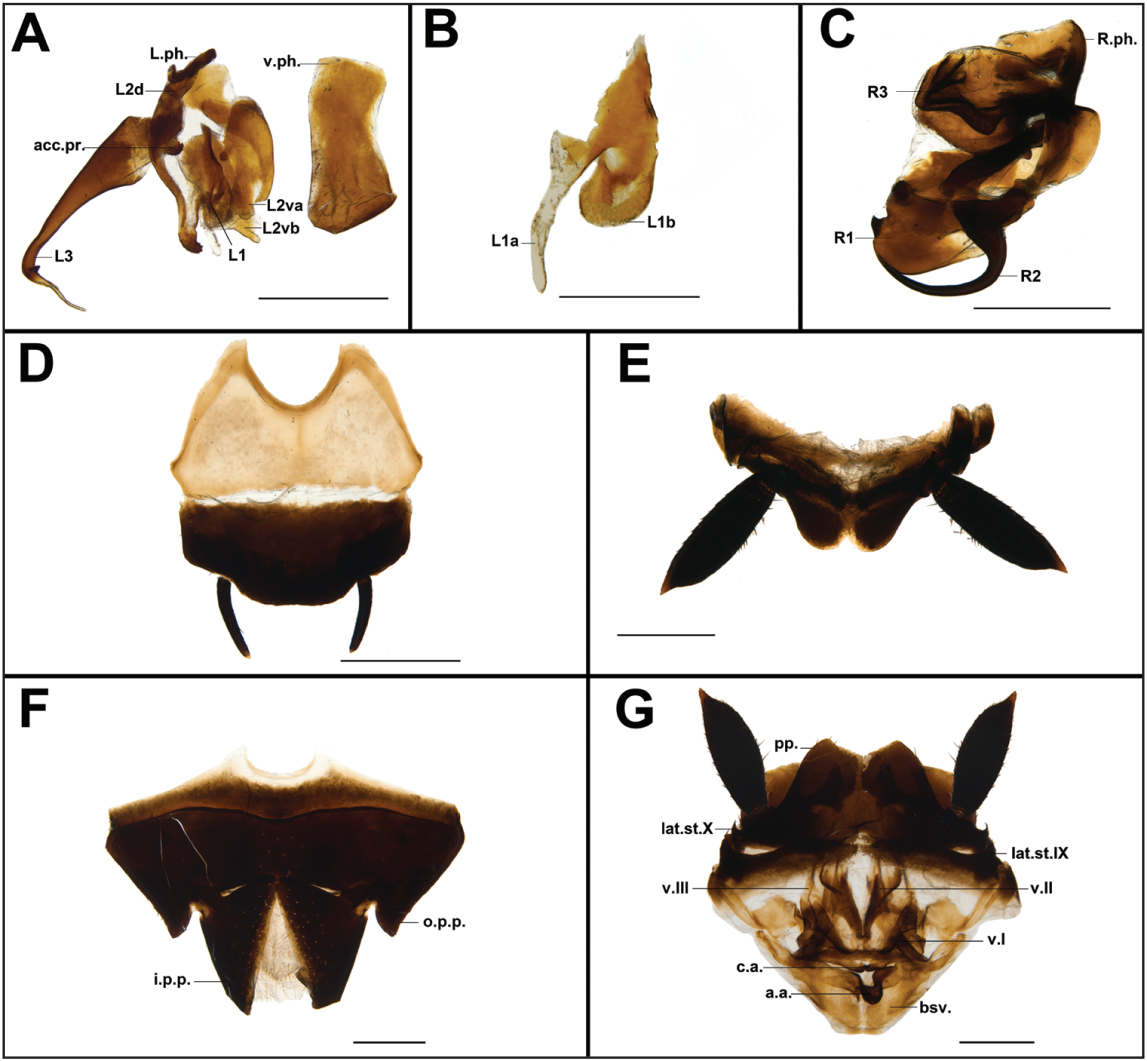
**A–G** male genitalia features from holotype **A** left phallomere, in dorsal view **B** L1 of left phallomere, in dorsal view **C** right phallomere, in dorsal view **D** subgenital plate, in ventral view **E** supra-anal plate, in dorsal view **F–G** female genitalia features from paratype **F** subgenital plate, in ventral view **G** supra-anal plate and genitalia, in dorsal view. Abbreviations: **a.a.**, anterior arch; **acc.pr.**, accessory process; **bsv.**, basivalvula; **c.a.**, central apodeme; **i.p.p.**, inner posterior process; **lat.st.IX–X**, laterosternal of the ninth-tenth segment; **L.ph.**, left phallomere; **L1–L3**, parts of left phallomere; **o.p.p.**, outer posterior process; **R.ph.**, right phallomere; **R1–R3**, parts of right phallomere; **v.I–III**, first-third valve; **v.ph.**, ventral phallomere. Scale bars: 1 mm (**A, C–G**); 0.5 mm (**B**).

**Figure 4.**
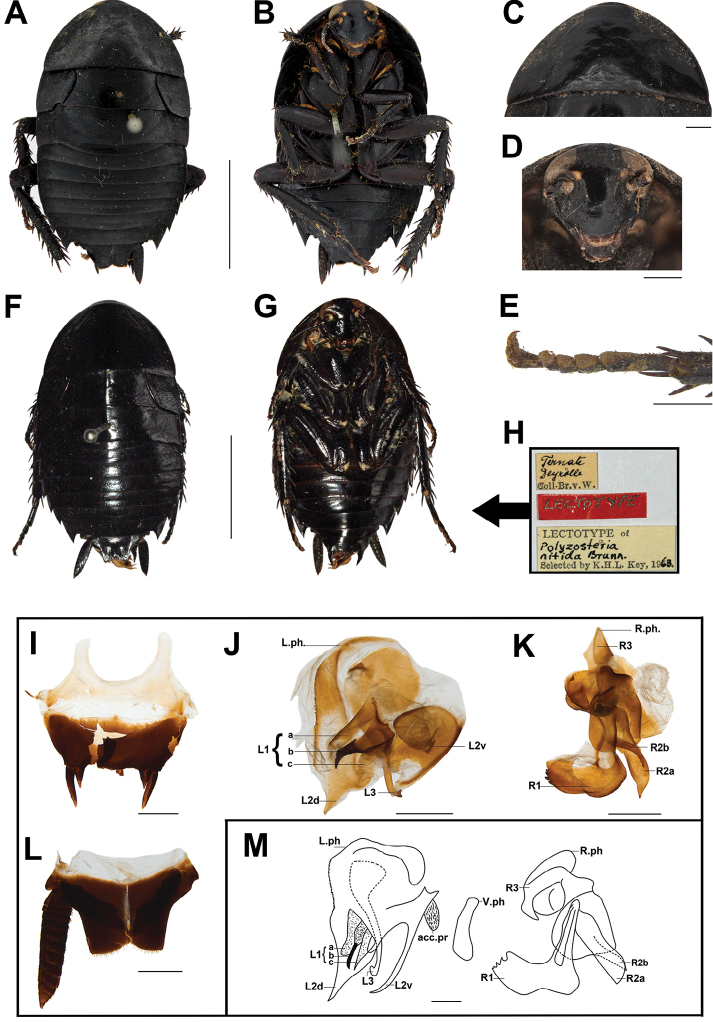
**A–E, I–L***Melanozosterianitida* from Guangxi, male **A** in dorsal view **B** in ventral view **C** pronotum, in dorsal view **D** head, in ventral view **E** tarsus, in ventral view **F–H** Lectotype of *Melanozosterianitida*, male **F** in dorsal view **G** in ventral view **H** labels **I** subgenital plate, in ventral view **J** left phallomere, in dorsal view **K** right phallomere, in dorsal view **L** supra-anal plate, in dorsal view **M** genitalia of *Melanozosterianitida* in Mackerras (1968a)**F–H** provided by H. Bruckner, Natural History Museum Vienna, NOaS Image Collection. Scale bars: 10 mm (**A–B, F–G**); 1 mm (**C–E, I–M**).

Based on former studies (Gutiérrez 2013, 2014; Mackerras 1965a, 1965b, 1965c, 1966a, 1966b, 1967a, 1967b, 1968a, 1968b; Rehn and Hebard 1927), the Polyzosteriinae is characterized as follows: species having semicircular pronotum, lobiform vestigial tegmina, angles of T2–T7 produced, tarsi usually short, bare or with hind and sometimes mid metatarsi spiny (*Laevifacies* with mid and hind metatarsi spiny, while in Mackerras (1968b), Australian species of Blattinae and Polyzosteriinae from other Blattidae with all metatarsi spiny), large pulvilli and arolia, cerci strong, short and symmetrical, L1 with hollow finger-like projection and sclerotized projection and R1 claw-like and margin with projection; thus, *Laevifacies* is placed in the subfamily Polyzosteriinae. *Laevifacies* has common features with *Melanozosteria*, *Eurycotis*, *Leptozosteria*, and *Platyzosteria*, such as body small to large, and shining, usually with vestigial tegmina, angles of T5–T6 acute, T6–T7 with punctation and hind metatarsus usually spiny (Gutiérrez 2013, 2014; Mackerras 1965c, 1968b). *Laevifacies* is similar to the *Melanozosteria* and *Eurycotis* in general appearance, but it can be distinguished from *Melanozosteria* by the following characters: 1) body thin and small in male (Figure 2A, B), while in *Melanozosteria*, it is broad and large (Figure 4A, B, F, G); 2) the surface of terga smooth (Figure 2A), vs. surface with punctation in *Melanozosteria* (Figure 4A, F); 3) male with vestigial tegmina and hind wings (Figure 2A), but in *Melanozosteria* only with vestigial tegmina or apterous (Figure 4A, F); 4) the margin of L2d smooth and posterior of L2d finger-like with more small spines (Figure 3A), while in *Melanozosteria* the margin strongly denticulate and posterior of L2d with acute angle (Figure 4J, M); 5) L3 bifurcated, one short and the other long (Figure 3A), however, L3 unbifurcated or bifurcated with branches of equal length in *Melanozosteria* (Figure 4J, M); 6) R1 fist-shaped (Figure 3C), while in *Melanozosteria* foot-shaped or finger-shaped (Figure 4K, M); and 7) R2 only with one large and long uniform structure (Figure 3C), while in *Melanozosteria*, two unequal forked structures present (Figure 4K, M); and it can be distinguished from *Eurycotis* by the following characters: 1) tibiae not specialized, while in *Eurycotis*, one group of which species have smooth surface, uniform black body and lateral tegmina, with highly specialized caudal tibiae; 2) R2 is hook-like, while in *Eurycotis* R2 is pincer-like. In addition, *Eurycotis* is restricted to South and North America and Cuba, while *Laevifacies* gen. nov. is found in East Asia. *Laevifacies* is similar to the *Methana* in the following genitalia characteristics, the margin of L2d smooth, R1 as a strongly claw-like sclerotized process, both of L1 have two structures, L1 of *Methana* has strong finger-like sclerotization and a membranous lobe, while *Laevifacies* has a finger-like membrane and a strongly sclerotized lobe (Figure 3A, B).

##### Geographical distribution.

China (Hainan).

#### Laevifacies quadrialata sp. nov.

Taxon classificationAnimaliaBlattodeaBlattidae

http://zoobank.org/CB699FB7-9F08-4830-A85D-948A8A48E629

##### Diagnosis.

Sexual dimorphism. Body small and black. Surface smooth and shining except last two terga with punctation. Tegmina and hind wings vestigial in male, tegmina vestigial and hind wings absent in female. Angles of T2–T7 sharp and protruded. Legs strong. Supra-anal plate short and triangular. Styli long and symmetrical.

##### Description.

**Measurements.** Male, pronotum: length × width 5.5–6.2 × 7.9–8.0 mm, overall length: 15.6–17.7 mm. Female, pronotum: length × width 7.0–7.1 × 10.5–10.7 mm, overall length: 17.0–21.0 mm.

Body black, smooth, shining. Vertex and frons black. Clypeus to part of labrum brown to dark brown, maxillary palpi and labial palpi dark brown to black. Eyes black when the specimens are fresh, fading after a long time (Figure 2D). Antennae dark brown with near middle segments and tip segments milky white (Figure 2A, B). Pronotum black, surface smooth and shining (Figure 2C). Tegmina and hind wings black, terga smooth except last two terga with punctation (Figure 2A, G). Sterna and legs dark brown to black. Cerci dark brown to black, apex yellowish brown (Figure 2A, B, G, H).

Size small to medium, female larger than male. Body oval, vertex nearly unexposed (Figure 2A, B, G, H). Ocelli present, small and round (Figure 2D). Pronotum nearly semicircular, anterior margin arc-shaped, posterior margin nearly straight, posterior angles blunt (Figure 2C). Small, vestigial tegmina and hind wings present in male, both extending to notal hind margin, only vestigial tegmina in female (Figure 2A, G); angles of T2–T7 sharp and protruded, sterna smooth and shining (Figure 2A, B, G, H). Legs strong, fore coxae with punctation; front femora Type A_2_ (Figure 2E); mid and hind metatarsus with a row of spines; hind metatarsus fairly long with pulvillus which occupies nearly one-quarter of its length, remainder of surface with hair, claws moderately symmetrical and unspecialized (Figure 2E, F, I, K). Male: supra-anal plate short, triangular, divided into two round lobes (Figure 3E); subgenital plate broad and short, posterior margin round; styli long and symmetrical (Figure 3D). Cerci symmetrical and strong, with indistinct segmentation, ends sharp (Figure 3E). Female: supra-anal plate with higher sclerotization (Figure 3G).

##### Male genitalia.

Left phallomere consisting of three parts: L1, L2, and L3. L1 with two parts L1a and L1b, L1a with membranous finger-like projection; L1b with sclerotized projection. L2 consisting of L2d and L2v, L2d strongly sclerotized in anterior part, the posterior part with finger-like and with more small spines; L2va simple and broad, L2vb sclerotized and the posterior with a spinous projection. L3 with a simple hook, elongate to the right and bifurcated (Figure 3A, B). Right phallomere consisting of R1, R2, and R3. R1 large, claw-like, right margin with a prominent spine; R2 large, curved hook-like, the base strong and gradually becoming thinner, bent to the right; R3 large and cucullate, highly sclerotized (Figure 3C).

##### Female genitalia.

The first valve (v.I) long, slightly broad and crescent-shaped, terminal membranous; the second valve (v.II) small, flaky and obscured by the v.I; the third valve (v.III) broader than v.I, terminal membranous; paraprocts (pp.) symmetrical and the middle concave; the middle of anterior arch (a.a.) concave; basivalvula (bsv.) trapezoidal (Figure 3G); inner posterior process of the laterosternal shelf (i.p.p.) divided in two parts, which are connected by hairy membrane; outer posterior process of the laterosternal shelf (o.p.p.) symmetrical, terminal with hairs (Figure 3F).

##### Material examined.

HOLOTYPE: male, CHINA, Hainan Prov., Baisha, Yinggeling Nature Reserve, 20-VIII-2010, Guo Zheng leg. PARATYPES: 1 male, same data as holotype; 1 male, Hainan Prov., Mt. Wuzhishan, 18-21-V-2014, Shunhua Gui, Xinran Li & Jianyue Qiu leg.; 1 male, Hainan Prov., Diaoluoshan, 18-IV-2015, Lu Qiu & Qikun Bai leg. (GenBank accession number: MK798103); 2 females, Hainan Prov., Lingshui, Mt. Diaoluoshan, 22-V-2014, Jianyue Qiu, Xinran Li & Shunhua Gui leg. (GenBank accession number: MK798104).

##### Etymology.

The species epithet comes from the Latin word *quadrialata* in reference to the male having four triangular vestigial wings.

##### Remarks.

In our study the interspecific K2P genetic divergence among *L.quadrialata* sp. nov. and other cockroach species ranged from 10.4 to 13.1%. But the genetic divergence value between male and female of *L.quadrialata* sp. nov. is only 0.9%, so we pair them based on their similar morphology combined with this COI data. Sexual dimorphism occurs in *L.quadrialata* sp. nov.: 1) females without hind wings, but males with vestigial hind wings (Figure 2A, 2G); 2) male with narrower body, while female with broader body (Figure 2A, B, G, H).

##### Geographical distribution.

China (Hainan)

### *Melanozosteria* Stål, 1874

*Melanozosteria* Stål, 1874: 13; Kirby 1904: 129; Shelford 1909: 265; Shelford 1910: 5 (as synonym of *Polyzosteria*); Princis 1966: 569; Mackerras 1968a: 237 (as subgenus); Roth 2003: 167; Rentz 2014: 151.

*Cutilia* Stål, 1877: 36; Kirby 1904: 134; Shelford 1909: 289; Shelford 1910: 7; Hanitsch 1915: 99; Princis 1949: 10.

*Symtomaptera* Tepper, 1893: 106 (as a subgenus of *Periplaneta*); Kirby 1904: 129; Shelford 1909: 265 (as a synonym of *Polyzosteria*); Princis 1949: 10 (as a synonym of *Melanozosteria*).

#### Melanozosteria nitida

Taxon classificationAnimaliaBlattodeaBlattidae

Brunner von Wattenwyl, 1865

##### Diagnosis.

Body broad oval and reddish brown to black. Pronotum slightly arched, surface with punctation. Vestigial tegmina sectorial with punctation, separated from mesonotum for nearly whole length, hind wings absent. Surface with punctation. Angles of T2–T7 protruded and sharp. The medial aspects to the styli with stubby and sharp spines.

##### Redescription.

##### Measurements.

Male, pronotum: length × width 7.4 × 12.5 mm, overall length: 26.1 mm.

Body uniformly deep reddish brown to black (Figure 4A, B, F, G). Eyes and ocelli yellowish white. Margin of clypeus and labrum dark brown. Vertex and frons black. Antennae brown or black, middle joints creamy-white (Figure 4D). Pronotum, tegmina, abdomen, legs and cerci all uniformly deep reddish brown to black (Figure 4C).

Body large, broad oval and convex, surface shining. Pronotum slightly arched, surface with punctation. Anterior margin of pronotum roundly protruded, and posterior margin straight (Figure 4C). Tegmina vestigial, sectorial, and separated from mesonotum, surface with punctation. Angles of metanotum protruded. Hind wings absent. Surface of all terga shining and with punctation; angles of T2–T7 protruded and sharp, T9 not protruded (Figure 4A, F). Legs short and thick. Fore coxae with slightly punctation; front femora Type A_2_ (anterior with two long spines, posterior with many small and slightly equal spines). Tibiae hair-brushes; hind tibiae with a row of spines, hind metatarsus with pulvillus occupying one-quarter to one-third of its length, remainder of ventral surface with spines (Figure 4E). All pulvilli large, claws symmetrical (Figure 4E). Supra-anal plate long, symmetrical and quadrilateral, side edge at gradient, angles of posterior round, the middle of posterior margin concave and with hair. Cerci thick, with blurry segmentation and the terminal segment spinous distally (Figure 4F, G, L). Subgenital plate nearly quadrilateral, short. The medial aspects to the styli with stubby and sharp spines (Figure 4I).

##### Male genitalia.

Left phallomere includes L1, L2, and L3. L1 with three parts (a, b, c). L1a slightly sclerotized, posterior not sclerotized, membranous and blunt. L1b more sclerotized and posterior sharp. L1c anterior slightly sclerotized and posterior blunt membrane. L2 includes L2d and L2v. L2d with a well-sclerotized, strongly denticulate in anterior margin, while the posterior of the sclerite becomes more delicate and ends in a sharp point; L2v usually single, L3 is a simple hook, but the posterior divides into two small forks which resemble an elephant’s nose (Figure 4J, 4M). Right phallomere includes R1, R2, and R3. R1 large, elongate, foot-like with broad down-turned “thumb” and 5–6 strongly denticulate on medial edge, R2a long, fairly broad, tapering slightly towards medial corner; R2b shorter, more strongly sclerotized and tapering to long narrow elongation. R3 with structure of folded sclerite (Figure 4K, 4M).

##### Materials examined.

1 male, CHINA, Guangxi Prov., Shangsi, Nadang, 15-XI-1958, Dexiang Gu & Jinting Liang leg.

##### Type specimen examined.

Lectotype of *Polyzosterianitida*, male, Ternate (Natural History Museum Vienna), “Ternate Jeynalle CoII. Br. V. W.”, “LECTOTYPE”, “LECTOTYPE of *Polyzosterianitida* Brunn. Selected by KHL Key, 1963.”; holotype of *Periplanetapolita*, male, Taiwan (Natural History Museum), “Holotype”, “*Periplanetapolita* Walker”, “BMNH (E) #878036”, presented by Beccaloni (2014).

##### Remarks.

We compared the lectotype of *M.nitida* (from Ternate, Indonesia) with the specimen from Guangxi and found there are minor differences between them: the styli are straight in the Guangxi individual (Figure 4I), but in the lectotype of *M.nitida*, slightly bent (Figure 4F, G). We also compared the genitalia between the Guangxi individual and the illustration in Mackerras (1968a); they share the typical characters of L1b spinous projection and serration along the margin of L2d, but they are also different in the following characteristics: 1) the terminal of L3 divided into two small forks, which resemble an elephant’s nose in the Guangxi individual (Figure 4J), while in the Mackerras (1968a) individual, L3 has one blunt hook (Figure 4M); 2) L2v broad and sclerotized, and posterior of L3 membranous in the Guangxi individual (Figure 4J), while L2v thin, long and with sharp sclerotized terminus in the Mackerras (1968a) individual (Figure 4M). And the variation of supra-anal plate between samples from Queensland and New Guinea were treated as intraspecific differences in different locations (Mackerras 1968a). Considering Mackerras (1968a) also recorded that the *M.nitida* is a widely distributed tropical species from Taiwan, Malaya, Moluccas, and Philippines, and due to our specimens being inadequate, the minor difference in the Guangxi individual and the lectotype of *M.nitida* are temporarily considered as the intraspecific differences of different populations.

##### Geographical distribution.

Australia, Philippines, Malaysia, New Guinea, New Caledonia, New Zealand, China, Thailand.

## Discussion

Almost all members in the Polyzosteriinae are brachypterous or apterous (excepting the tribe Methanini), and display high developmental stochasticity (Rentz 2014). The Australian Polyzosteriinae exhibit the best examples of aposematic coloration. They are often being metallically colored, or spotted and barred with bright orange, red, or yellow markings (Rentz 1996; Roach and Rentz 1998). When disturbed, they may first display a warning signal before resorting to defensive measures (Bell et al. 2007). However, *Laevifaciesquadrialata* sp. nov. did not attract our attention due to their bland appearance and life in a hidden habitat (usually hidden in bushes, Lu Qiu, pers. obs.), even with sexual dimorphism. Sexual dimorphism is very common in cockroaches, some of which beinng so extreme that it is a challenge for taxonomists to match the two sexes (Roth 1992). In this study, sexual dimorphism is revealed for the first time in Polyzosteriinae on the basis of COI data, and exhibits mainly in the body size and the vestigial hind wings.

## Supplementary Material

XML Treatment for Laevifacies

XML Treatment for Laevifacies quadrialata

XML Treatment for Melanozosteria nitida
